# *Streptomyces aridus* sp. nov., isolated from a high altitude Atacama Desert soil and emended description of *Streptomyces noboritoensis* Isono et al. 1957

**DOI:** 10.1007/s10482-017-0838-2

**Published:** 2017-02-09

**Authors:** Hamidah Idris, David P. Labeda, Imen Nouioui, Jean Franco Castro, Maria del Carmen Montero-Calasanz, Alan T. Bull, Juan A. Asenjo, Michael Goodfellow

**Affiliations:** 1 School of Biology, Newcastle University, Ridley Building 2, Newcastle upon Tyne, NE1 7RU UK; 2 National Center for Agricultural Utilization Research, USDA-ARS, Peoria, IL 61604 USA; 3 Department of Chemical Engineering and Biotechnology, Centre for Biotechnology and Bioengineering (CeBiB), University of Chile, Beauchef 851, Santiago, Chile; 4 School of Biosciences, University of Kent, Canterbury, Kent CT2 7NJ UK

**Keywords:** *Streptomyces*, *Aridus*, Polyphasic taxonomy, Atacama Desert

## Abstract

A polyphasic study was undertaken to determine the taxonomic status of a *Streptomyces* strain which had been isolated from a high altitude Atacama Desert soil and shown to have bioactive properties. The strain, isolate H9^T^, was found to have chemotaxonomic, cultural and morphological properties that place it in the genus *Streptomyces*. 16S rRNA gene sequence analyses showed that the isolate forms a distinct branch at the periphery of a well-delineated subclade in the *Streptomyces* 16S rRNA gene tree together with the type strains of *Streptomyces crystallinus, Streptomyces melanogenes* and *Streptomyces noboritoensis*. Multi-locus sequence analysis (MLSA) based on five house-keeping gene alleles showed that isolate H9^T^ is closely related to the latter two type strains and to *Streptomyces polyantibioticus* NRRL B-24448^T^. The isolate was distinguished readily from the type strains of *S. melanogenes, S. noboritoensis* and *S. polyantibioticus* using a combination of phenotypic properties. Consequently, the isolate is considered to represent a new species of *Streptomyces* for which the name *Streptomyces aridus* sp. nov. is proposed; the type strain is H9^T^ (=NCIMB 14965^T^=NRRL B65268^T^). In addition, the MLSA and phenotypic data show that the *S. melanogenes* and *S. noboritoensis* type strains belong to a single species, it is proposed that *S. melanogenes* be recognised as a heterotypic synonym of *S. noboritoensis* for which an emended description is given.

**Electronic supplementary material:**

The online version of this article (doi:10.1007/s10482-017-0838-2) contains supplementary material, which is available to authorized users.

## Introduction

Streptomycetes remain a unique source of novel pharmaceutically important products with antibacterial, anti-inflammatory and antitumor activities (Bérdy 2005; Demain 2014; Barka et al. 2016), hence the continued interest in these organisms as a source of new specialised metabolites (Goodfellow and Fiedler 2010; Chaudhary et al. 2013). However, the continued search for new bioactive compounds against drug-resistant microorganisms needs to be focused on novel streptomycetes to avoid the rediscovery of known compounds from common *Streptomyces* species. Given this imperative, novel *Streptomyces* species are being sought from unusual and neglected habitats (Hong et al. 2009; Tiwari and Gupta 2012), notably from extreme biomes (Bull 2011; Hamedi et al. 2013; Goodfellow 2013). Previous work from our group has shown that novel *Streptomyces* species abound in arid Atacama Desert soils (Okoro et al. 2009; Busarakam 2014; Busarakam et al. 2014), some of which synthesise new specialised metabolites with encouraging bioactivities (Bull et al. 2016), thereby underpinning the premise that extreme environmental conditions promote unique actinobacterial diversity which is the basis of novel chemistry (Bull and Stach 2007; Bull 2011; Gomez-Escribano et al. 2015).

*Streptomyces*, the type genus of the family *Streptomycetaceae,* was proposed by Waksman and Henrici (1943) and the description of the taxon emended by Witt and Stackebrandt (1990) and Wellington et al. (1992). The genus encompasses aerobic, Gram-stain positive actinobacteria with a high DNA G+C content, which form extensively branched substrate mycelia supporting aerial hyphae that typically differentiate into chains of spores, have a wall peptidoglycan rich in LL-diaminopimelic acid, contain major amounts of saturated, iso- and anteiso- fatty acids, usually have either hexa- or octa-hydrogenated menaquinones with nine isoprene units as predominant isoprenologues and complex polar lipid patterns which tend to include diphosphatidylglycerol, phosphatidylethanolamine, phosphatidylinositol and phosphatidylinositol mannosides (Kämpfer 2012). The genus includes over 700 validly named species (http://www.bacterio.net/streptomyces.html) which can be assigned to many multi- and single-membered subclades in the *Streptomyces* 16S rRNA gene tree (Kämpfer 2012; Labeda et al. 2012) and based on multi-locus sequence analysis (MLSA; Labeda et al. 2017). Despite being the largest genus in the domain *Bacteria* the taxon remains underspeciated (Okoro et al. 2009; Antony-Babu et al. 2010; Busarakam 2014). New species are assigned to the genus using a combination of genotype and phenotype properties, and some recent studies have featured MLSA (Rong and Huang 2012, 2014; Labeda et al. 2014, 2017; Labeda 2016).

The aim of the present study was to establish the taxonomic position of a *Streptomyces* strain, isolate H9^T^, which had been isolated from a high altitude Atacama Desert soil and shown to produce novel specialised metabolites (Idris 2016). The results of a polyphasic taxonomic study showed that isolate H9^T^ belongs to a novel *Streptomyces* species for which we propose the name *Streptomyces aridus* sp. nov.

## Materials and methods

### Selective isolation, maintenance and cultural conditions

Strain H9^T^ was recovered from an arid subsurface soil sample (30 cm depth) at 4000 metres above sea level on Cerro Chajnantor (23^o^63′31″S, 67^o^52′27″W) east of San Pedro de Atacama, Chile. The strain was isolated on glucose-yeast extract agar (Athalye et al. 1981) supplemented with cycloheximide and nystatin (each at 25 µg/ml) after incubation at 28 °C for 14 days following inoculation with a suspension of one gram of soil in 5 ml ¼ strength Ringer’s solution. The isolate together with *S. melanogenes* NRRL B-2072^T^, *S. noboritoensis* NRRL B-12152^T^ and *S. polyantibioticus* NRRL B-24448^T^ were maintained on yeast extract-malt extract agar (International *Streptomyces* Project [ISP medium 2], Shirling and Gottlieb 1966). All of the reference strains were obtained from the NRRL culture collection as indicated.

Biomass for the chemotaxonomic and molecular systematic analyses was prepared in shake flasks (180 revolutions per minute) of ISP2 broth following incubation at 28 °C for 14 days and washed twice in distilled water; cells for the chemotaxonomic studies were freeze-dried and those for the molecular systematic analyses stored at room temperature.

### Chemotaxonomy

Strain H9^T^ was examined for chemotaxonomic properties known to be characteristic of *Streptomyces* strains (Kämpfer 2012). Standard procedures were used to detect isomers of diaminopimelic acid (A_2_pm) (Staneck and Roberts 1974), menaquinones (Collins et al. 1985), polar lipids (Minnikin et al. 1984) and whole cell sugar composition (Lechevalier and Lechevalier 1970), using appropriate controls. The type strains of *S. melanogenes* and *S. noboritoensis* were also included in the analyses for A_2_pm isomers, cellular sugars and polar lipids. Fatty acid methyl esters (FAMEs) were prepared from isolate H9^T^ and the type strains of *S. melanogenes, S. noboritoensis* and *polyantibioticus* by saponification, methylation and extraction following protocols developed by Miller (1982) with minor modifications from Kuykendall et al. (1988). The FAMEs were separated by gas chromatography (Agilent 6890 N instrument) and the resultant peaks automatically integrated. Fatty acid names and properties were determined using the standard microbial identification (MIDI) system Version 4.5 and the ACTIN 6 database (Sasser 1990).

### Phylogenetic analyses

#### 16S rRNA gene sequencing

Genomic DNA was extracted from isolate H9^T^ biomass and PCR-mediated amplification of a 16S rRNA purified gene product obtained, as described by Kim and Goodfellow (2002). Identification of phylogenetic neighbours and calculation of pairwise 16S rRNA gene sequence similarities were realised using the EzTaxon-e server (http://eztaxon-e.ezbiocloud.net/; Kim et al. 2012). The CLUSTAL W algorithm from MEGA 6 (Tamura et al. 2013) was used to align the sequences. Phylogenetic trees were generated using the maximum-likelihood, maximum-parsimony and neighbour-joining algorithms drawn from the MEGA 6 software package. Evolutionary distances were calculated using the Kimura two-parameter (Kimura 1980) and the topologies of the resultant trees evaluated by bootstrap analyses (Felsenstein 1985) based on 1000 replicates. The trees were rooted using the 16S rRNA gene sequence of *Streptomyces albus* subsp*. albus* DSM 40313^T^ (GenBank accession number AJ 621602).

#### Multi-locus sequence analyses

The experimental and data handling procedures used in the MLSA were based upon modifications of described procedures (Labeda et al. 2014, 2017; Labeda 2016). Genomic DNA was isolated from the strain using an UltraClean^®^ Microbial DNA isolation kit (MoBio Labs, Carlsbad, CA) by following the manufacturer’s instructions. Partial sequences of the house-keeping genes *atpD* (ATP synthase F1, beta subunit), *gyrB* (DNA gyrase B subunit), *rpoB* (RNA polymerase beta subunit), *recA* (recombinase A) and *trpB* (tryptophan synthetase, beta subunit) were amplified and sequenced using primers and protocols, as described previously by Labeda et al. 2014. The amplified products were purified using ExoSAP-IT (Affymetrix, Santa Clara, CA), sequenced using BigDye 3.1 on an ABI sequencer model 3730 and assembled using Sequencher version 5.2 (Gene Codes, Ann Arbor, MI).

The gene sequences for the 5 house-keeping loci of the strain were deposited in GenBank (see Supplemental Table S1) and were also organised using the Bacterial Isolate Genomic Sequence Database (BIGSdb) version 1.12.3 (Jolley and Maiden 2010) on the ARS Microbial Genomic Sequence Database server (http://199.133.98.43). Genome sequences, where available, were uploaded into the sequence bin for the respective isolates in the BIGSdb isolate database. The genome sequences were scanned within BIGSdb for house-keeping loci, the sequences of which were then tagged and the allele sequences and respective allele designations added to the sequence database when new alleles were found. The strain record was then updated with the matching allele identification for each locus held in the strain database. The sequences for the alleles of the loci of isolate H9^T^ were individually aligned with MAFFT (Katoh and Standley 2013), subsequently concatenated head to tail in-frame, and exported in FASTA format, providing a dataset of 706 *Streptomyces* strains and 2622 positions (Labeda 2016).

Phylogenetic relationships were constructed in IQ-Tree version 1.41 (Nguyen et al. 2015) using the maximum-likelihood algorithm based on the general time reversible model (Nei and Kumar 2000 with invariable sites plus a discrete Gamma-model based on 4 rate categories (Gu et al. 1995) which had been shown to be the optimal model for such data using iModelTest 2 (Darriba et al. 2012). The individual trees were the subject of 1000 ultrafast bootstrap replications (Minh et al. 2013) followed by 1000 replications of assessment of branch supports with single branch tests using the SH-like approximate likelihood ration test (Guindon et al. 2010). MLSA evolutionary distances were determined using MEGA 6 by calculating the Kimura 2-parameter distances (Kimura 1980). Strain pairs having ≤0.007 MLSA evolutionary distances were considered conspecific based on the guideline empirically determined by Rong and Huang (2012), namely that this MLSA distance (Kimura 2-parameter distance), computed from the partial sequences of these house-keeping loci, equates to the 70% DNA:DNA cut-off point recommended for the delineation of prokaryotic species by Wayne et al. (1987).

### Cultural and morphological properties

The cultural features of isolate H9^T^ and the type strains of *S. melanogenes, S. noboritoensis* and *S. polyantibioticus* were recorded on tryptone-yeast extract, yeast extract-malt extract, oatmeal, inorganic salts-starch, glycerol-asparagine, peptone-yeast extract-iron and tyrosine agar plates (ISP media 1–7, Shirling and Gottlieb 1966) that had been incubated for 14 days at 28 °C. Spore chain morphology and spore surface ornamentation were detected following growth on oatmeal agar (ISP medium 3; Shirling and Gottlieb 1966) for 14 days at 28 °C, by scanning electron microscopy (Cambridge 240 instrument) after O’Donnell et al. (1993).

### Phenotypic tests

Strain H9^T^ and the type strains of the three reference *Streptomyces* species were examined for a range of standard biochemical, degradative and physiological properties using media and methods described by Williams et al. (1983). Enzyme profiles of the strains were determined using API ZYM kits (bioMérieux) following the manufacturer’s instructions. A standard inoculum corresponding to 5 on the McFarland scale (Murray et al. 1999) was used to inoculate all of these tests. In addition, the ability of the strains to oxidise diverse carbon and nitrogen sources and to show resistance to inhibitory compounds were determined using GEN III microplates in an Omnilog device (BIOLOG Inc., Haywood, USA). The exported data were analysed using the opm package for R (Vaas et al. 2012, 2013) version 1.06. All of these tests were carried out in duplicate.

## Results and discussion

**Fig. 1:**
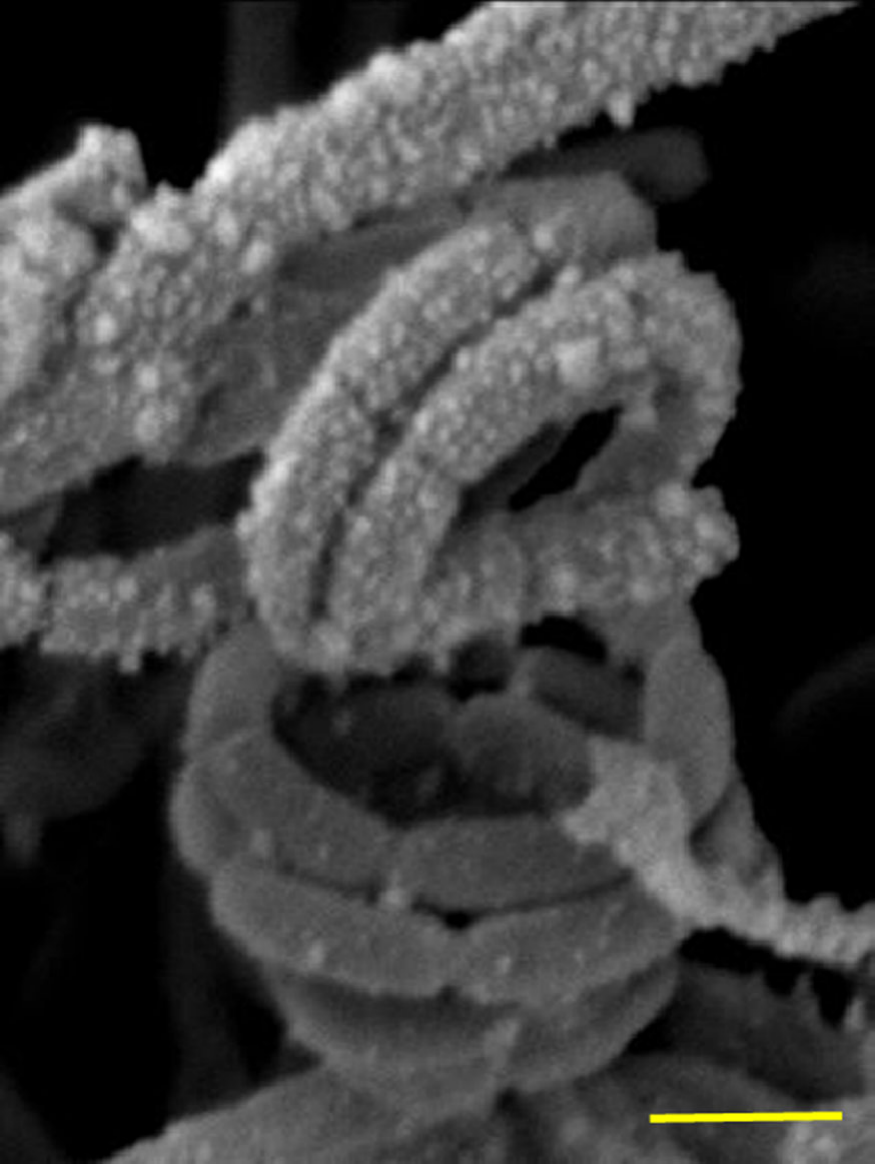
Scanning electron micrograph of isolate H9^T^ showing spiral chains of smooth surfaced spores following growth on oatmeal agar at 28 °C for 10 days. Bar 1 µm

The chemotaxonomic, cultural and morphological properties of strain H9^T^ were found to be consistent with its classification in the genus *Streptomyces* (Kämpfer 2012). The organism forms an extensively branched substrate mycelium which carries aerial hyphae that differentiate into spiral chains of smooth surfaced spores on all of the ISP media tested, as exemplified in Fig. 1. The strain forms brown to black substrate mycelia on most of the ISP media and either mild brown or brown black diffusible pigments on ISP media 5–7 (Table 1). Whole organism hydrolysates of the strain were found to be rich in LL-A_2_pm, glucose, mannose and ribose; the predominant fatty acids were identified as anteiso-C_15:0_ (34.6%), iso-C_16:0_ (19.4%), anteiso-C_17:0_ (17.9%) and C_16:0_ (10.9%) (Table 2); the major isoprenologues were identified as tetra-, hexa- and octa-hydrogenated menaquinones (16, 23 and 30%, respectively); and the polar lipid pattern was found to consist of diphosphatidylglycerol, phosphatidylethanolamine, phosphatidylglycerol, phosphatidylinositol, glycophosphatidylinositol, one unidentified lipid and an aminolipid (Fig. S1).

**Table 1:** Growth and cultural characteristics of *Streptomyces* isolate H9^T^ and the type strains of *S. melanogenes, S. noboritoensis* and *S. polyantibioticus* on ISP media after 14 days at 28 °C

Characteristic	ISP						
	1	2	3	4	5	6	7
Isolate H9^T^							
Growth	++	+++	++	++	+++	++	++
Aerial spore mass	None	None	White	White (edge) and pale yellow pink (middle)	Light brown grey	None	None
Substrate mycelium	Brown black	Brown black	Dark grey brown	Brown grey	Brown black	Dark olive brown	Black
Diffusible pigments	None	None	None	None	Mild brown	Mild brown	Brown black
*S. melanogenes*							
Growth	++	++	+++	++	+	+	+
Aerial spore mass	None	None	White	Grey yellow brown	None	Light grey yellow brown	None
Substrate mycelium	Dark grey yellow brown	Dark yellow brown	Dark yellow brown	Light grey yellow brown	Mild yellow brown	Dark grey yellow brown	Deep yellow brown
Diffusible pigments	Deep yellow brown	None	None	None	None	Dark yellow brown	None
*S. noboritoensis*							
Growth	++	+++	+++	+++	+++	++	+
Aerial spore mass	None	Grey yellow brown	Yellow white	White	None	None	None
Substrate mycelium	Mild yellow brown	Dark grey yellow brown	Mild yellow brown	Dark grey yellow brown	Dark grey yellow brown	Dark grey yellow brown	Dark grey yellow brown
Diffusible pigments	None	None	None	None	None	None	None
*S. polyantibioticus*							
Growth	++	+++	+++	+++	+++	++	+
Aerial spore mass	None	White	White	Light grey	White	None	None
Substrate mycelium	Dark orange yellow	Dark orange yellow	Mild yellow brown	Light grey yellow brown	Dark yellow brown	Dark yellow brown	Drk orange yellow
Diffusible pigments	None	None	None	None	None	None	None

+++ abundant growth, ++ very good growth, + poor growth

**Table 2:** Fatty acid profiles (%) of *Streptomyces* isolate H9^T^ and the type strains of *Streptomyces melanogenes, Streptomyces noboritoensis and Streptomyces polyantibioticus*

Fatty acid	Isolate H9	*S. melanogenes* NRRL B- 2072^T^	*S. noboritoensis* NRRL B-12152^T^	*S. polyantibioticus* NRRL B-24448^T^
C_12:0_	–	0.1	–	–
C_13:0_	–	0.1	–	–
*anteiso*-C_13:0_	–	0.2	–	0.1
*iso*-C_13:0_	0.1	0.3	0.1	0.2
C_14:0_	0.1	0.4	0.3	0.3
*iso*-C_14:0_	5.7	2.3	4.2	1.1
C_15:0_	0.3	2.3	0.6	3.1
*anteiso*- C_15:0_	34.6	24.1	21.6	31.2
*iso*-C_15:0_	2.1	12.3	15.4	10.4
C_15:0_ *ω*6c	–	0.1	–	0.1
*iso*- C_16:0_	19.4	12.7	19.4	9.8
*iso*- H C_16:0_	–	0.2	4.5	0.2
Summed feature 3	0.6	1.3	0.9	1.3
C_16:0_	10.9	11.7	8.6	10.0
C_16:1_ *ω*9c	–	1.0	–	–
*iso*-C_17:0_ *ω*9c	0.7	2.1	2.6	1.8
*anteiso*-C_17:0_ *ω*9c	0.3	1.6	1.7	0.8
*iso*-C_17:0_	5.7	9.6	8.6	9.3
*anteiso*-C_17:0_	17.9	15.0	9.9	19.7
C_17:1_ *ω*8c	0.2	0.1	–	0.8
C_17:0_	0.5	1.4	0.4	2.3
C_17:0_ 2OH	–	0.1	–	–
*iso*-C_18:0_	0.8	0.3	0.4	0.2
C_18:0_	0.2	0.1	–	0.2
C_18:0_ *ω*9c	–	0.1	–	–

– fatty acid not detected; Summed feature 3: 16:1 *ω*7c/15 *iso* 2 OH

**Fig. 2:**
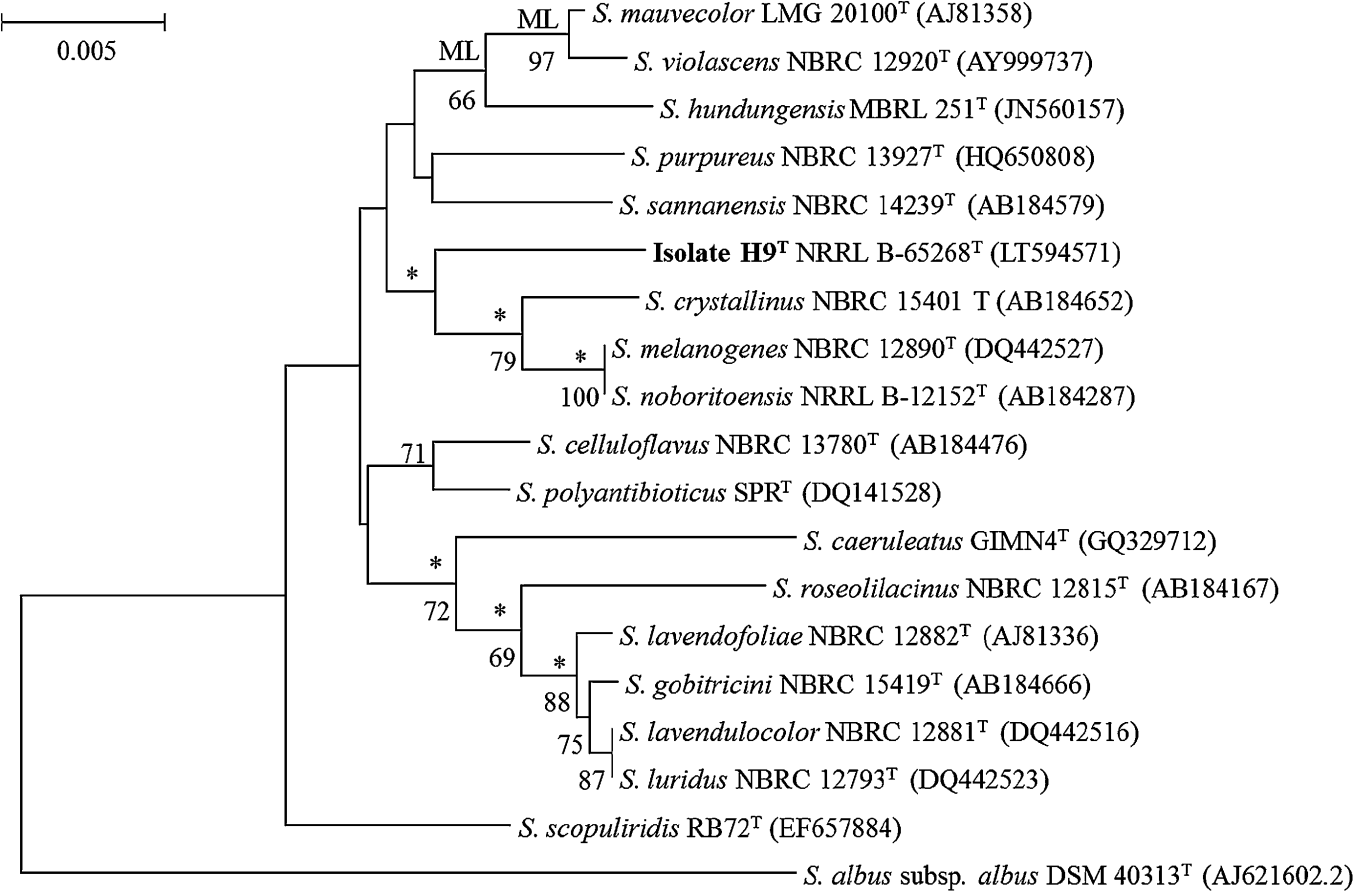
Neighbour-joining phylogenetic tree based on nearly complete 16S rRNA gene sequences (1329–1425 nucleotides) showing relationships between isolate H9^T^ and closely related type strains of *Streptomyces* species. Asterisks indicate branches of the tree that were recovered using the maximum-likelihood (ML) and maximum-parsimony tree-making methods. ML indicates branches of the tree that were also supported by this algorithm. Numbers at the modes indicate levels of bootstrap support based on a neighbour-joining analysis of 1000 resampled datasets, only values above 50% are given. The scale bar indicates 0.005 substitutions per nucleotide position

Isolate H9^T^ was found to form a distinct branch at the periphery of a well delineated subclade in the *Streptomyces* 16S rRNA gene tree together with *S. crystallinus* NBRC 15401^T^, *S. melanogenes* NBRC 12890^T^ and *S. noboritoensis* NRRL B-12152^T^, a relationship that was supported by all of the tree-making algorithms but not by a high bootstrap value (Fig. 2). The isolate is closely related to the *S. melanogenes* and *S. noboritoensis* strains sharing a 16S rRNA gene sequence similarity with them of 98.6%, a value found to correspond to 20 nucleotide (nt) differences at 1424 and 1423 locations respectively; the *S. melanogenes* and *S. noboritoensis* strains were shown to have identical 16S rRNA gene sequences. A subclade consisting of *S. melanogenes* NBRC 12890^T^ and *S. noboritoensis* NRRL B-12152^T^ was identified as cluster 30 in the 16S rRNA gene analysis of Labeda et al. (2012), *S. polyantibioticus NRRL B*-*24448* ^*T*^ was found adjacent to this taxon. The corresponding 16S rRNA gene sequence homologies between the isolate and the remaining phylogenetically close strains were found to fall within the range 97.7–98.5%, values shown to equate to 21 and 31 nt differences.

**Fig. 3:**
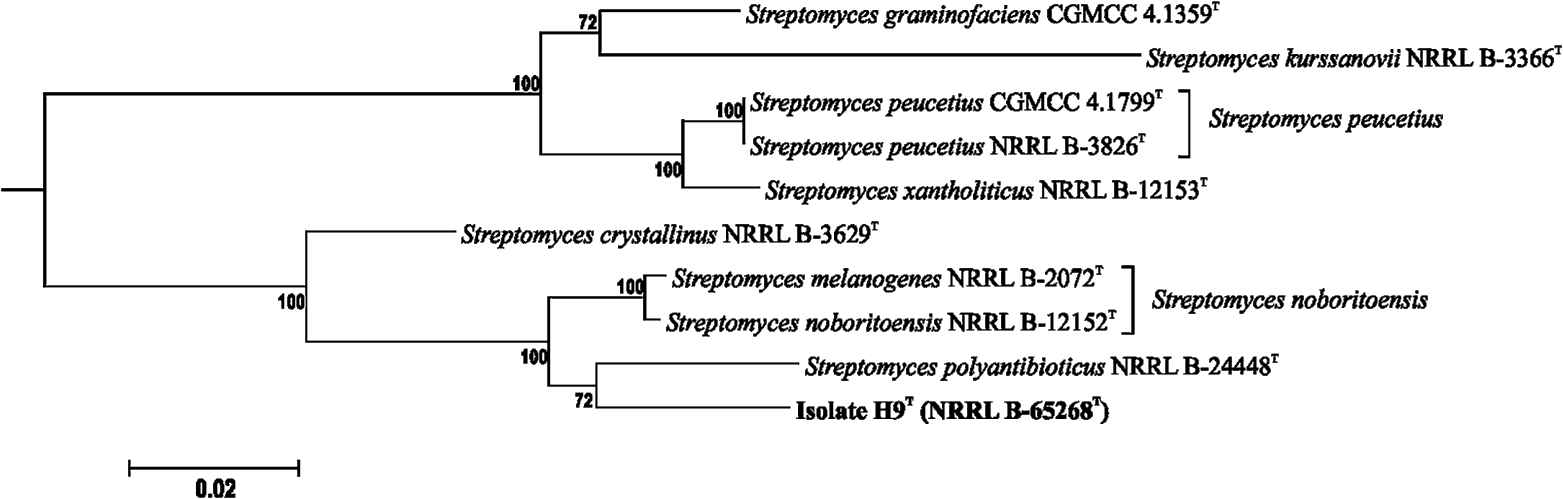
*Streptomyces* sub-tree derived from the phylogenetic tree inferred from concatenated partial sequences of the house-keeping genes *atpD, gyrB, recA, rpoB* and *trpB* using the maximum-likelihood method based on the General Time Reversible model. The final dataset consisted of 2622 positions and 706 strains. Percentages at the nodes represent levels of bootstrap support from 1000 resampled datasets, values less than 60% are not shown. The proposed new species is indicated in bold. Bar, equals number of substitutions per site

MLSA have been found to clarify relationships between closely related streptomycetes because of the strong phylogenetic signal provided by partial sequences of single-copy house-keeping genes (Rong and Huang 2012, 2014; Labeda 2011, 2016, Labeda et al. 2014, 2016, 2017); In the present MLSA analysis, the relationships found between isolate H9^T^ and the type strains of closely related *Streptomyces* species are shown in Fig. 3 and Table 3. The isolate is closely related to *S. melanogenes* NRRL B-2072^T^, *S. noboritoensis* NRRL B-1252^T^ and *S. polyantibioticus* NRRL B-24448^T^, relationships that are supported by a 100% bootstrap value; *S. crystallinus* NRRL B-3629^T^ is loosely associated with this lineage. The relationship of *S. crystallinus* NBRC 15401^T^, *S. melanogenes* NBRC 12890^T^ and *S. noboritoensis* NRRL B-12152^T^ was also identified in a recent MLSA study (Labeda et al. 2017). Isolate H9^T^ was shown to have MLSA distances greater than 0.007 with all of these strains indicating that it forms the nucleus of a novel *Streptomyces* species. In contrast, the type strains of *S. melanogenes* and *S. noboritoensis* share a MLSA evolutionary distance of only 0.004 indicating that they belong to the same genomic species.

**Table 3:** MLSA distances for strains phylogenetically near to isolate H9^T^ and related isolates

	Strain	MLSA (Kimura 2-paramenter) distance									
		1	2	3	4	5	6	7	8	9	10
1	*S. halstedii* CGMCC 4.1359^T^	–									
2	*S. kurssanovii* NRRL B-3366^T^	0.063									
3	*S. peucetius* CGMCC 4.1799^T^	0.040	0.073								
4	*S. peucetius* NRRL B-3826^T^	0.040	0.073	0.000							
5	*S. xantholiticus* NRRL B-12153^T^	0.038	0.076	0.014	0.014						
6	*S. crytalllinus* NRRL B-3629^T^	0.094	0.123	0.093	0.093	0.097					
7	*S. melanogenes* NRRL B-2072^T^	0.112	0.142	0.109	0.109	0.114	0.049				
8	*S. noboritoensis* NRRL B-12152^T^	0.113	0.142	0.106	0.106	0.111	0.048	0.004			
9	*S. polyantibioticus* NRRL B-24448^T^	0.110	0.139	0.107	0.107	0.110	0.060	0.035	0.035		
10	*Streptomyces* species H9^T^ (NRRL B-65268^T^)	0.111	0.138	0.107	0.107	0.110	0.054	0.034	0.034	0.039	–

Identical results were obtained for nearly all of the duplicated phenotypic tests, the exceptions being a few carbon source tests recorded from the GEN111 microplates. It can be seen from Table 4 that the isolate can be distinguished from the type strains of *S. melanogenes, S. noboritoensis* and *S. polyantibioticus*, its close phylogenetic neighbours, by a broad range of phenotypic tests though all four strains have many properties in common. In particular, the isolate can be distinguished from the three reference type strains by its ability to form spiral chains of spores, grow at 40 °C and oxidise d-fucose and d-raffinose. Conversely, the three reference type strains can be differentiated from isolate H9^T^ by their ability to form straight to flexuous spore chains and degrade arbutin, hypoxanthine and l-tyrosine. In turn, the isolate can be distinguished from *S. melanogenes* NRRL B-2072^T^ and *S. noboritoensis* NRRL B-12151^T^ by its ability to degrade casein, starch and Tween 80, but not arbutin, guanine or urea.

**Table 4:** Phenotypic properties that differentiate *Streptomyces* isolate H9^T^ from the type strains of *Streptomyces melanogenes*, *Streptomyces noboritoensis* and *Streptomyces polyantibioticus*

Phenotypic tests	Isolate H9^T^	*S. melanogenes* NRRL B- 2072^T^	*S. noboritoensis* NRRL B-12152^T^	*S. polyantibioticus* NRRL B-24448^T^
Morphology				
Spore chains	Spiral	Straight to flexuous^a^	Straight to flexuous^b^	Spores held within sporangia^c^
API ZYM tests				
α-Chymotrypsin	–	–	–	+
Esterase (C4)	+	–	+	+
β-Galactosidase	+	+	–	+
α-Glucuronidase	+	+	–	+
GEN III BIOLOG microplates				
Oxidation of:				
*N*-acetyl-d-Galactosamine, d-fructose, inosine, d-mannose	+	+	+	–
l-Arginine	–	–	+	+
d-Aspartic acid, *N*-acetyl-β- d-mannosamine	–	+	–	+
Citric acid	–	+	+	+
d-Fructose-6-phosphate	–	+	–	+
d-Fucose, d-raffinose	+	–	–	–
Guanidine	–	–	+	–
β-hydroxy-Butyric acid	+	–	+	+
β-methyl-d-Glucoside	–	+	–	–
Pectin	+	–	–	–
l-Pyroglutamic acid	+	–	–	+
d-Salicin	–	–	–	+
d-Turanose	+	+	–	–
Inhibition tests				
Sodium bromate	+	+	+	–
Sodium lactate (1%)	–	–	–	+
Tetrazolium blue	–	–	+	–
Growth in the presence of				
Sodium chloride (4%, w/v)	+	+	+	–
Growth at				
pH 5	–	–	–	+
Other phenotypic tests				
Biochemical tests				
Allantoin hydrolysis	–	–	–	+
Urea hydrolysis	–	+	+	–
Degradation tests				
Arbutin	–	+	+	+
Casein	+	–	–	+
Elastin	–	+	+	+
Guanine	–	+	+	–
Hypoxanthine, l-tyrosine	–	+	+	+
Starch	+	–	–	+
Uric acid	+	+	+	–
Tween 80	+	–	–	+
Growth at				
40 °C	+	–	–	–

+, positive result; −, negative result

^a^^,^^b^ and ^c^, data taken from Isono et al. (1957) and (le Roes-Hill and Meyers 2009), respectively

Positive results recorded for *Streptomyces* isolate H9^T^, *S. melanogenes* NRRL B- 2072^T^, *S. noboritoensis* NRRL B-12152^T^ and *S. polyantibioticus* NRRL B-24448^T^

*API ZYM tests*: acid phosphatase, alkaline phosphatase, cystine arylamidase, esterase lipase (C8), β-glucosidase, leucine arylamidase, lipase (C14), α-mannosidase, naphthol-AS-BI-phosphohydrolase, *N*-acetyl-β-glucosaminidase and valine arylamidase

*GEN III BIOLOG microplates*: utilisation of l-alanine, l-aspartic acid, l-glutamic acid, l-histidine, l-serine (amino acids), γ-amino-*n*-butyric acid, α-*keto*-butyric acid, α-*keto*-glutaric acid, acetic acid acetoacetic acid, d-gluconic acid, l-malic acid, propionic acid (organic acids); glycyl-proline (peptide); d-cellobiose, dextrin, l-fucose, d-galactose, 3-*O*-methyl-d-galactose, β-gentiobiose, d-glucose, glycerol, d-melibiose, stachyose (sugars); d-trehalose (sugar alcohol) and growth in the presence of potassium tellurite, rifamycin SV and sodium chloride (1%, w/v)

Other phenotypic tests: aesculin hydrolysis, degradation of adenine and Tween 40 and growth at 10, 20 and 30 °C

Negative results recorded for *Streptomyces* isolate H9^T^, *S. melanogenes* NRRL B- 2072^T^, *S. noboritoensis* NRRL B-12152^T^ and *S. polyantibioticus* NRRL B-24448^T^

*API ZYM tests*: α-fucosidase, α-galactosidase, β-glucuronidase and trypsin

*GEN III BIOLOG microplates*: utilisation of d-serine#1, d-serine#2, (amino acids); butyric acid, d-malic acid, mucic acid, *N*-acetyl-neuraminic acid, quinic acid, d-saccharic acid, (organic acids); α-d-lactose, l-rhamnose, stachyose (sugars); d-galacturonic acid, l-galactonic acid-γ-lactone, (sugar acids); d-arabitol, d-mannitol, d-salicin, d-sorbitol (sugar alcohols); glucuronamide (amino hexose) and resistance to fusidic acid, guanidine hydrochloride, lincomycin, minocycline, niaproof, sodium formate, tetrazolium violet, tetrazolium blue, troleandomycin, vancomycin and growth in the presence of sodium chloride (8%, w/v) and at pH 6

*Other phenotypic tests*: H_2_S production, nitrate reduction, degradation of cellulose, chitin, xanthine, xylan and tributyrin and growth at 4 or 45 °C

It is apparent that the type strains of *S. melanogenes* and *S. noboritoensis* have many phenotypic properties in common, notably morphological and cultural features found to be of particular value in the circumscription of *Streptomyces* species by Labeda et al. (2012) in their phylogenetic survey of *Streptomycetaceae* species. The present results are in good agreement with those of previous studies in which the type strains of *S. melanogenes* and *S. noboritoensis* were assigned to the same numerically defined phenotypic clusters (Williams et al. 1983; Kämpfer et al. 1991) and the same MLSA lineage (Labeda et al. 2017). In addition, all three strains were shown to have whole organism hydrolysates rich in LL-A_2_pm, glucose, mannose and ribose and similar polar lipid patterns (Fig S1). Similar chemotaxonomic markers have been recorded for the type strain of *S. polyantibioticus,* NRRL B-2443^T^ (Roes and Meyers 2009). The fatty acid profiles of the type strains of *S. melanogenes*, *S. noboritoensis* and S. *polyantibioticus,* like that of isolate H9^T^, were shown to contain major proportions of anteiso-C_15:0_, iso-C_16:0_, C_16:0_ and anteiso-C_17:0_ though quantitative differences were apparent; such differences were also detected between other fatty acids though a few trace components were discontinuously distributed (Table 2).

In summary, isolate H9^T^ is only loosely associated with its near phylogenetic neighbours in the *Streptomyces* 16S rRNA gene tree and its distinctness is strongly supported by corresponding MLSA data based on concatenated sequences of five house-keeping genes. It can also be distinguished from the type strains of *S. melanogenes* and *S. noboritoensis*, its close phylogenetic neighbours, based on a combination of phenotypic properties. These data clearly show that isolate H9^T^ forms a new centre of taxonomic variation within the genus *Streptomyces*. The name proposed for this taxon is *Streptomyces aridus* sp. nov. It is also clear from the MLSA and associated chemotaxonomic and phenotypic data that the type strains of *S. melanogenes* and *S. noboritoensis* belong to the same species. It is, therefore, proposed that *S. melanogenes* Suguwara and Onuma (1957) be seen as a heterotypic synonym of *S. noboritoensis* Isono et al. (1957). An emended description is given of the latter.

### Description of *Streptomyces aridus* sp. nov.

*Streptomyces aridus* (*a’ri. dus.* L. masc. adj. *aridus,* dry, referring to the isolation of the strain from arid soil).

Aerobic, Gram-stain positive, catalase positive actinobacterium that forms an extensively branched substrate mycelium that bears aerial hyphae that differentiate into spiral chains of smooth surfaced spores (1–1.5 µm × 0.5 µm) on oatmeal agar. A brown–black diffusible pigment is produced on yeast extract-malt extract agar. Grows from 10 to 40 °C, optimally ~28 °C, from pH 5–10, optimally ~pH 7.0 and in the presence of up to 2.5% w/v NaCl. Additional cultural and phenotypic features are cited in the text and in Tables 1 and 4. Chemotaxonomic characteristics are typical of the genus *Streptomyces.* The type strain, H9^T^ (=NCIMB 14965^T^=NRRL B-65268^T^), was isolated from a subsurface soil sample collected at 4000 metres above sea level on Cerro Chajnantor, east of San Pedro de Atacama in north eastern Chile. The GenBank accession number for the 16S rRNA gene sequence of isolate H9^T^ is LT594571.

### Emended description of *Streptomyces noboritoensis* Isono et al. 1957, 21^AL^

Heterotypic synonym: *Streptomyces melanogenes* Suguwara and Onuma 1957, 141^AL.^

Data taken from the present study and from Kämpfer (2012).

Aerobic, Gram-stain positive actinobacterium which forms extensively branched substrate mycelia that bear aerial hyphae which differentiate into straight to filamentous spore chains. Mature spore chains are long with 10–50, or often more than 50, spores per chain. This morphology is seen on glycerol-asparagine agar, oatmeal agar, salts-starch agar and yeast extract-malt extract agar. Spore surface is smooth. Grows at 10, 20 and 30 °C but not at 4 or 40 °C, at pH6 and pH7 and in the presence of 4%, w/v sodium chloride. Additional cultural and phenotypic properties are cited in the text and in Tables 1 and 4. Chemotaxonomic features are typical of the genus *Streptomyces.*

The type strain of *S. noboritoensis* (NRRL B-12152^T^), was isolated from soil from Inada-noborito, Kawasaki City, Kanagawa Prefecture, Japan.

Type strain: ATCC 23937, CBS 92168, DSM 40192, NBRC 12390, JCM 4378, NCIMB 9835, NRRL B-2072, RIA 1146.

## Electronic supplementary material

Below is the link to the electronic supplementary material.

Supplementary material 1 (DOCX 721 kb)

Supplementary material 2 (XLSX 11 kb)
