# Supplementary material for: Streptomyces aridus sp. nov., isolated from a high altitude Atacama Desert soil and emended description of Streptomyces noboritoensis Isono et al. 1957
Source: Antonie Van Leeuwenhoek. 2017 Feb 9;110(5):705–17. doi: 10.1007/s10482-017-0838-2 (PMC5387016; doi:10.1007/s10482-017-0838-2)
Supplement: Supplementary file 1 — Supplementary material 1 (DOCX 721 kb) [file 10482_2017_838_MOESM1_ESM.docx]

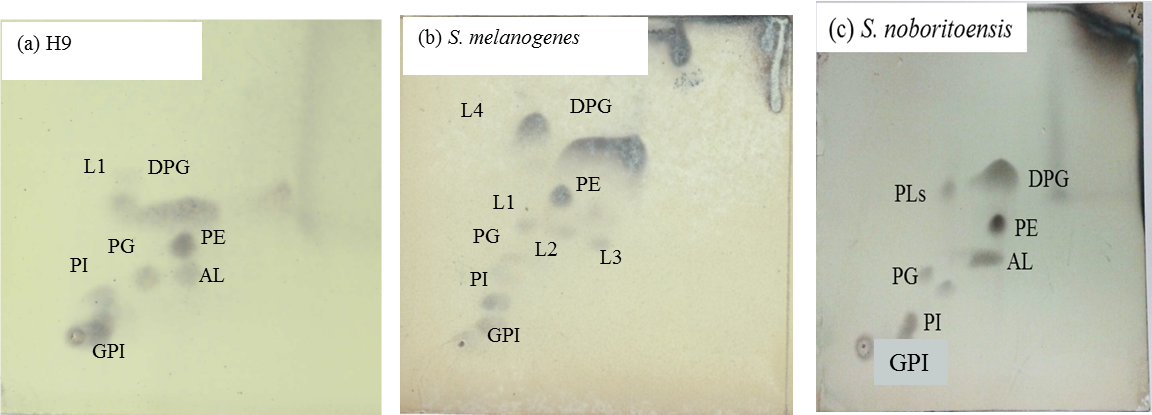


Figure S1..Two-dimensional thin-layer chromatography of polar lipids of (a) isolate H9^T^, (b) *Sterptomyces melanogenes* NRRL B-2072^T^ and (c) *Streptomyces noboritoensis* NRRL B-12152^T^ stained with molybdatophosphoric acid spray (Sigma P1518) Abbreviations: DPG, diphosphatidylglycerol; PE, phosphatidylethanolamine; GPI, glycophosphatidylinositol; PG, phosphatodylglycerol, PI, phosphatidylinositol; PL, phospholipids; AL, aminolipid; and L, unidentified lipids.
